# Inter- and Intrapopulational Heterogeneity of Characteristic Markers in Adult Human Neural Crest-derived Stem Cells

**DOI:** 10.1007/s12015-021-10277-w

**Published:** 2021-11-08

**Authors:** Beatrice A. Windmöller, Anna L. Höving, Cornelius Knabbe, Johannes F. W. Greiner

**Affiliations:** 1 Department of Cell Biology, University of Bielefeld, Bielefeld, Germany; 2 Forschungsverbund BioMedizin Bielefeld FBMB e.V, Bielefeld, Germany; 3 Present Address: Department of Cellular Neurophysiology, Faculty of Medicine, University of Bielefeld, Bielefeld, Germany; 4 Institute for Laboratory and Transfusion Medicine, Heart and Diabetes Centre NRW, Ruhr-University Bochum, 32545 Bad Oeynhausen, Germany

**Keywords:** Heterogeneity, Adult human stem cells, Neural crest-derived stem cells, Inferior turbinate stem cells, Stochastic heterogeneity, Clonal heterogeneity

## Abstract

**Abstract:**

Adult human neural crest-derived stem cells (NCSCs) are found in a variety of adult tissues and show an extraordinary broad developmental potential. Despite their great differentiation capacity, increasing evidence suggest a remaining niche-dependent variability between different NCSC-populations regarding their differentiation behavior and expression signatures. In the present study, we extended the view on heterogeneity of NCSCs by identifying heterogeneous expression levels and protein amounts of characteristic markers even between NCSCs from the same niche of origin. In particular, populations of neural crest-derived inferior turbinate stem cells (ITSCs) isolated from different individuals showed significant variations in characteristic NCSC marker proteins Nestin, S100 and Slug in a donor-dependent manner. Notably, increased nuclear protein amounts of Slug were accompanied by a significantly elevated level of nuclear NF-κB-p65 protein, suggesting an NF-κB-dependent regulation of NCSC-makers. In addition to this interpopulational genetic heterogeneity of ITSC-populations from different donors, single ITSCs also revealed a strong heterogeneity regarding the protein amounts of Nestin, S100, Slug and NF-κB-p65 even within the same clonal culture. Our present findings therefor strongly suggest ITSC-heterogeneity to be at least partly based on an interpopulational genetic heterogeneity dependent on the donor accompanied by a stochastic intrapopulational heterogeneity between single cells. We propose this stochastic intrapopulational heterogeneity to occur in addition to the already described genetic variability between clonal NCSC-cultures and the niche-dependent plasticity of NCSCs. Our observations offer a novel perspective on NCSC-heterogeneity, which may build the basis to understand heterogeneous NCSC-behavior.

**Graphical Abstract:**

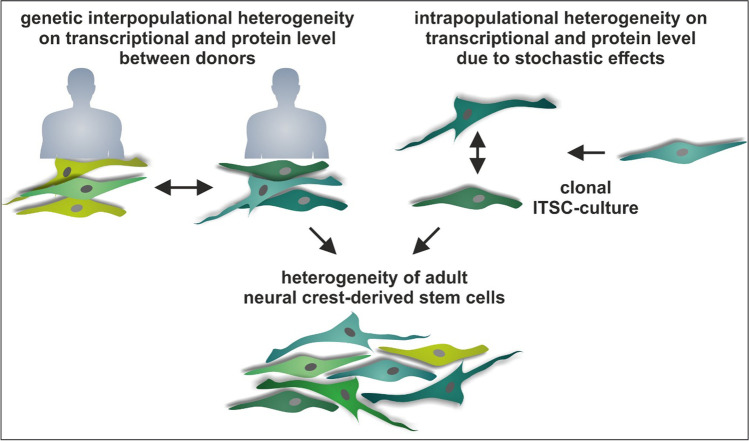

## Introduction

Stem cells with an embryonic origin from the neural crest crucially participate in embryonic development but are also commonly known to remain within the organism until adulthood, where they reside as quiescent adult stem cells. During development, the neural crest (NC) arises between the future ectoderm and the neural tube following the process of neurulation [1]. After undergoing epithelial to mesenchymal transition (EMT), neural crest cells (NCCs) migrate in a range of developing tissues and contribute to development by guiding patterning and by differentiation. Amongst others, NCCs give rise to most parts of the craniofacial skeleton, enteric neurons of the foregut and stomach or sympathetic ganglia and melanocytes in the trunk (reviewed in [2–4]). After participating to embryonic development, pools of NCCs remain as adult neural crest-derived stem cells (NCSCs) in various tissues including the skin [5], hair follicles [6], carotid body [7], heart [8, 9], palatum [10] or nasal cavity [11–13] (reviewed in [2, 3]). Adult NCSCs share a broad differentiation potential particularly into mesodermal and ectodermal derivates as well as the expression of characteristic markers. These marker proteins include the intermediate filament Nestin and the calcium binding protein S100 as well as NC-related transcription factors (TF) like Twist, Snail or Slug, which belong to group of TF enabling EMT and therefore migration of embryonic NCCs (reviewed in [2, 3]). Despite similarities in differentiation capacity and marker expression, increasing evidences show a remaining heterogeneity between different NCSC-populations (reviewed in [2]). In this regard, we very recently observed an interpopulational heterogeneity between craniofacial and cardiac NCSCs. Although we demonstrated high similarities between the global transcriptomes of both NCSC-populations, gene expression patterns and differentiation potentials differed with particular regard to their tissue of origin [8]. Here, cardiac stem cells showed an exclusively high capacity for differentiation into cardiomyocytes, while craniofacial NCSCs more efficiently gave rise to neurons or osteoblasts compared to their cardiac counterparts [8]. These observations are in line with several studies reporting an association between the differentiation behaviors of NCSCs and their niche. For instance, NCSCs from the murine carotid body were demonstrated not only to undergo neurogenesis but even angiogenesis *in vivo *[7, 14]. On the contrary, NC-derived dental pulp stem cells showed the capacity to form dentin and dental pulp tissue in addition to their neuronal and osteogenic differentiation potential [15], suggesting a niche-dependent plasticity of adult NCSCs.

In the present study, we extended this perspective on NCSC-heterogeneity by reporting a highly heterogeneous marker expression between and even within NCSC-populations from the same niche of origin. We particularly took advantage of an adult human craniofacial NCSC-population isolated from the inferior turbinate of the nasal cavity (inferior turbinate stem cells, ITSCs) [13], which we previously utilized for the interpopulational comparison with cardiac NCSCs [8]. Like other NCSC-populations, ITSCs show a broad differentiation potential including the capacity to give rise to glutamatergic and dopaminergic neurons [16, 17] or osteoblasts [18] as well as the expression of common NCSC-markers [13, 19]. However, our present data reveal a broad heterogeneity between ITSCs regarding the expression and protein amounts of such NCSC-markers, particularly Nestin, S100, Twist and Slug. On an interpopulational level, the protein amounts of Nestin, S100 and Slug significantly differed between ITSC-populations from distinct donors. Notably, significantly elevated nuclear protein levels of Slug in one female donor were accompanied by a significantly increased amount of nuclear factor kappa-light-chain-enhancer of activated B cells (NF-κB) protein in ITSCs. NF-κB is a key regulator of various cellular processes including cell growth, inflammation, memory or immunity [20–22] and was described to control inflammatory responses and fate choices of ITSCs [23, 24]. As for Nestin, S100 and Slug, the amount of nuclear NF-κB-p65 protein within a population of ITSCs was found to be highly dependent on the donor. In addition to this interpopulational heterogeneity, we observed a remarkable intrapopulational heterogeneity of Nestin, S100, Slug and NF-κB-p65 protein levels between single ITSCs within a respective population and even within clonally grown cultures.

## Materials and Methods

### Cell Culture

ITSCs were isolated from inferior turbinate tissue obtained during routine nasal surgery according to Hauser and coworkers [13] after an informed consent according to local and international guidelines. All experimental procedures were ethically approved by the ethics board of the medical faculty of the University of Münster (No. 2012–015-f-S). Experiments were conducted using ITSCs from 6 male donors and 5 female donors (Table 1).

**Table 1:** Overview of all ITSC-Donors included within this study

Sex and number of ITSC donor	Age at sampling
Male donor I	58 years old
Male donor II	43 years old
Male donor III	45 years old
Male donor IV	49 years old
Male donor V	34 years old
Male donor VI	49 years old
Female donor I	21 years old
Female donor II	67 years old
Female donor III	61 years old
Female donor IV	26 years old
Female donor V	40 years old

Cells were cultured in Dulbecco’s modified Eagle’s medium/Ham F-12 (DMEM-F12) (Sigma Aldrich, St. Louis, MO, USA) supplemented with 40 ng/ml basic fibroblast growth factor-2 (FGF2) (Miltenyi Biotec, Bergisch Gladbach, Germany), 20 ng/ml epidermal growth factor (EGF) (Miltenyi Biotec), 1x B27-Supplement (Thermo Fisher Scientific, Waltham, MA, USA), 20 mM L-glutamine (Sigma Aldrich), 10 mg/ml Penicillin/Streptomycin (Sigma Aldrich) and 0,25 mg/ml Amphotericin B (Sigma Aldrich) with additional 10 % human blood plasma (Institute for Laboratory- and Transfusion- Medicine, Heart- and Diabetes-Centre NRW, Bad Oeynhausen, Germany) at 37 °C, 5 % O_2_ and 5 % CO_2_ in a humidified incubator (Binder, Germany) as previously described [19]. Passaging was performed using Collagenase I (Sigma Aldrich) as previously described [13, 19]. Clonal culture of ITSCs was performed as described before [25]. Briefly, enzymatically dissociated cells were diluted in culture medium to obtain 1 cell per 100 µl. 100 µl of cell suspension were subsequently placed into 96-well plates and assessment of successfully plated single cells was performed 4 h later.

### Transfection of Cultivated ITSCs

ITSCs cultivated as described above were enzymatically detached using Collagenase I and harvested at 300 x g for 10 min. Dissociated cells were transfected with 1 µg pmax GFP vector (Amaxa Biosystems, Switzerland) and Amaxa rat NSC-Nucleofector Kit (Amaxa Biosystems) and the Nucleofector II device (Amaxa Biosystems) according to the manufacturer’s information. 5 ml DMEM F-12 (Sigma Aldrich) were added before the sample was centrifuged for 10 min at 300 x g. After discarding the supernatant, cells were cultured as described above. Imaging of transfected cells was performed using a Axio Observer.D1-Microscope (Carl Zeiss AG, Oberkochen, Germany).

### Flow Cytometry and FACS

Flow cytometric analysis and fluorescence activated cell sorting (FACS) of GFP-transfected ITSCs was performed using CyFlow Space (Partec, Münster, Germany). 10,000 GFP^+^ cells were sorted and collected in 15 ml reaction tubes (Sarstedt, Nümbrecht, Germany) and subsequently stored on ice for further experimental procedures. Single cells were obtained for further transcriptomic analyses by serial dilution (see below). Data were processed using FlowJo software (Tree Star, OR, USA).

### Serial Dilution

To isolate single cells, a solution of FACS-sorted GFP^+^ ITSCs was diluted serially in phosphate buffered saline (PBS) (Sigma Aldrich) to obtain 1 cell / 0.5 µl. Subsequently 0.5 µl of the diluted cell suspension was placed into one well of 96-well microtiter plates and the presence of a single cell was verified by fluorescence microscopy.

### SMARTseq2

After the isolation of single cells via serial dilution (see above), SMARTseq2 was performed according to Picelli and colleagues [26]. Briefly, cells were transferred in a total volume of 0.5 µl into 2 µl of cell lysis buffer consisting of 0.2 % (v/v) Triton X-100 (Sigma Aldrich) and 2 U/µl RNase Inhibitor (Invitrogen, Carlsbad, CA, USA) with additionally 1 µl oligo-dT primers and 1 µl of dNTP mix (NEB Biolabs, Ipswich, MA, USA). Further, hybridization of the oligo-dT primers to the poly (A) tail of the mRNA, first-strand-synthesis with template switching and PCR-preamplification were performed as described by Picelli and coworkers [26].

### RT-PCR

PCR was performed using GoTaq DNA polymerase (Promega, Fitchburg, WI, USA) according to the manufacturer’s guidelines with the following primers for eEF2 (AGGTCGGTTCTACGCCTTTG, TTCCCACAAGGCACATCCTC), Vimentin (GTGGACCAGCTAACCAACGACAAA, AGGTCAGGCTTGGAAACATCCACA), GAPDH (CATGAGAAGTATGACAACAGCCT, AGTCCTTCCACGATACCAAAGT), S100 (GGGAGACAAGCACAAGCTGAAGA, TCAAAGAACTCGTGGCAGGCAGTA), Snail (CCCAATCGGAAGCCTAACTA, GGACAGAGTCCCAGATGAGC) and Twist (GTCCGCAGTCTTACGAGGAG, CCAGCTTGAGGGTCTGAATC), Nestin (CGCACCTCAAGATGTCCCTC, CAGCTTGGGGTCCTGAAAGC), Slug (TCGGACCCACACATTACCTT, TTGGAGCAGTTTTTGCACTG).

### Immunocytochemistry

For immunocytochemistry, ITSCs cultivated as described above were seeded on top of cover glasses and cultivated for two days followed by fixation with 4 % paraformaldehyde (lab-made) in phosphate-buffered saline for 15 min at room temperature (RT). Permeabilization and blocking was subsequently done using 0,02 % Triton X-100 (Sigma-Aldrich, Germany) and 5 % goat serum (Dianaova, Germany) or 1 % BSA (in PBS) diluted for 30 min at RT. Primary antibodies against Nestin (Mouse, 10C2, 1:300, Merk KGaA, Germany), S100 (Rabbit, Z0311, 1:400, Dako Cytomation, Germany), Slug (Rabbit, C19G7, 1:100, Cell Signaling, Germany) or NF-κB-p65 (Mouse, 200,301,065, 1:5000, Rockland, PA USA) were incubated for 1 h at RT. Secondary fluorochrome-conjugated antibodies (Alexa 555 anti-mouse, Alexa 555 anti-rabbit, Alexa 488 antimouse, 1:300, Life Technologies, Germany) were incubated for 1 h at RT followed by nuclear counterstaining with DAPI (1 µg/ml in 1 x PBS; Sigma-Aldrich, Germany) for 10 min at RT. Fluorescence imaging was done with a confocal laser scanning microscope (LSM 780; Carl Zeiss, Jena, Germany). Five randomly placed pictures were taken per donor and analyzed using ImageJ/Fiji [27]. Measurements of fluorescence intensity were performed using the “measure” function in ImageJ/Fiji after selecting the regions of interest (cytosol or nucleus).

### Statistical Analysis

Data were raised at least in biological triplicates and were statistically analyzed using the Prism V5.01 software (GraphPad Software, Inc., San Diego, CA, USA). Test for normality was conducted using D’Agostino and Pearson omnibus normality test. To evaluate differences between multiple groups, we performed the Kruskal-Wallis test and as post test the Dunn’s Multiple Comparison Test. A significance value of p < 0.05 was considered as statistically significant. The data are presented as means ± standard error of the mean (SEM).

## Results

### Transcriptional Profiling of Single Adult Human Inferior Turbinate Stem Cells Reveals Strong Differences in the Expression of NC-specific Genes

**Fig. 1:**
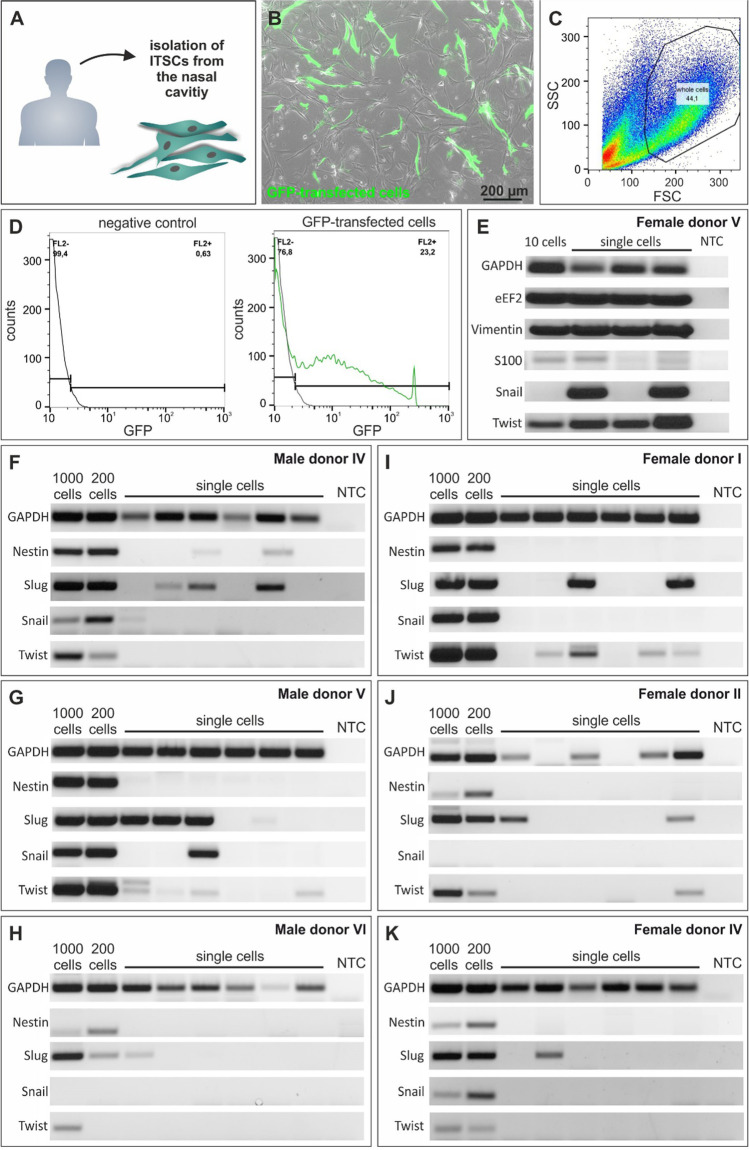
SMARTseq2 reveals strong transcriptional heterogeneity of single ITSCs in their NCSC-marker expression. **A**: ITSCs were derived from the inferior turbinate of the human nasal cavity. **B**: GFP-transfected ITCSs 24 h after transfection. **C**: Flow cytometric analyses of ITSCs. SSC and FSC measure size and granularity of the cells. Whole cells (44.1 %) are marked for further gating. **D**: Sorting of GFP^+^ ITSCs. FL2 measures emitted fluorescence signal (GFP). Untransfected cells served as negative control. **E**: RT-PCR after SMARTseq2 for the housekeeping genes GAPDH, eEF2 and Vimentin as well as for the NCSC-markers S100, Snail and Twist. **F**-**K**: RT-PCRs after application of SMARTseq2 on manually diluted single cells from three male and three female donors for the housekeeping gene GAPDH and the stem cell and neural crest markers Nestin, Slug, Snail and Twist

To initially assess the potential heterogeneity of inferior turbinate stem cells isolated from the human nasal cavity (Fig. 1A) on transcriptional level, we successfully applied SMARTseq2 [26] on single ITSCs. To facilitate a fluorescence-based sorting procedure, ITSCs were transfected with green fluorescent proteint (GFP) (Fig. 1B). Analysis of GFP^+^-cells during flow cytometric sorting revealed a transfection efficiency of 23.2 % (Fig. 1C, D). SMARTseq2 was performed with samples comprising ten cells and three samples consisting of one cell each. Following RT-PCR analysis revealed the presence of transcripts for GAPDH, eEF2 and Vimentin in the ten-cells-sample as well as in all three single cell approaches (Fig. 1E). Further transcriptional profiling revealed the heterogeneous expression of the NCSC-markers S100, Snail and Twist, the latter being key players in EMT and highly important in the determination of NCSC-fate (reviewed in [2]). In particular, S100 gene expression was detectable in the 10-cell-sample and in two out of three single-cell-samples. Interestingly, Snail transcripts could not be observed within the 10-cell-sample but in two of the single ITSCs. Notably, Twist mRNA could be successfully amplified in the sample originating from ten ITSCs as well as in all three single-cell-samples with varying expression level (Fig. 1E). These data indicate a general transcriptional heterogeneity between single ITSCs from the same population. This assumption was confirmed by further RT-PCR assays after the application of SMARTseq2 on six manually diluted single ITSCs from three male and female donors each (Fig. 1F-K). RT-PCR analysis exhibited the presence of the housekeeping gene GAPDH in samples comprised of 1000 and 200 cells as well as in almost all single cell samples. Transcriptional profiling of the stem cell and neural crest markers Nestin, Slug, Snail and Twist depicted strong heterogeneity in the expression of these genes for single ITSCs from the same donor as well as for single ITSCs from different donors in a sex independent manner. Additionally, there was no consistent NCSC-marker-expression-pattern observable for the investigated single cells, suggesting the presence of stochastic fluctuations in gene expression (Fig. 1F-K). Conclusively, we detected strong transcriptional heterogeneity of single ITSCs between the investigated donors as well as for single ITSCs from the same donor.

### ITSCs Show Highly Heterogeneous Protein Levels of Nestin and S100 in a Donor-dependent Manner

**Fig. 2:**
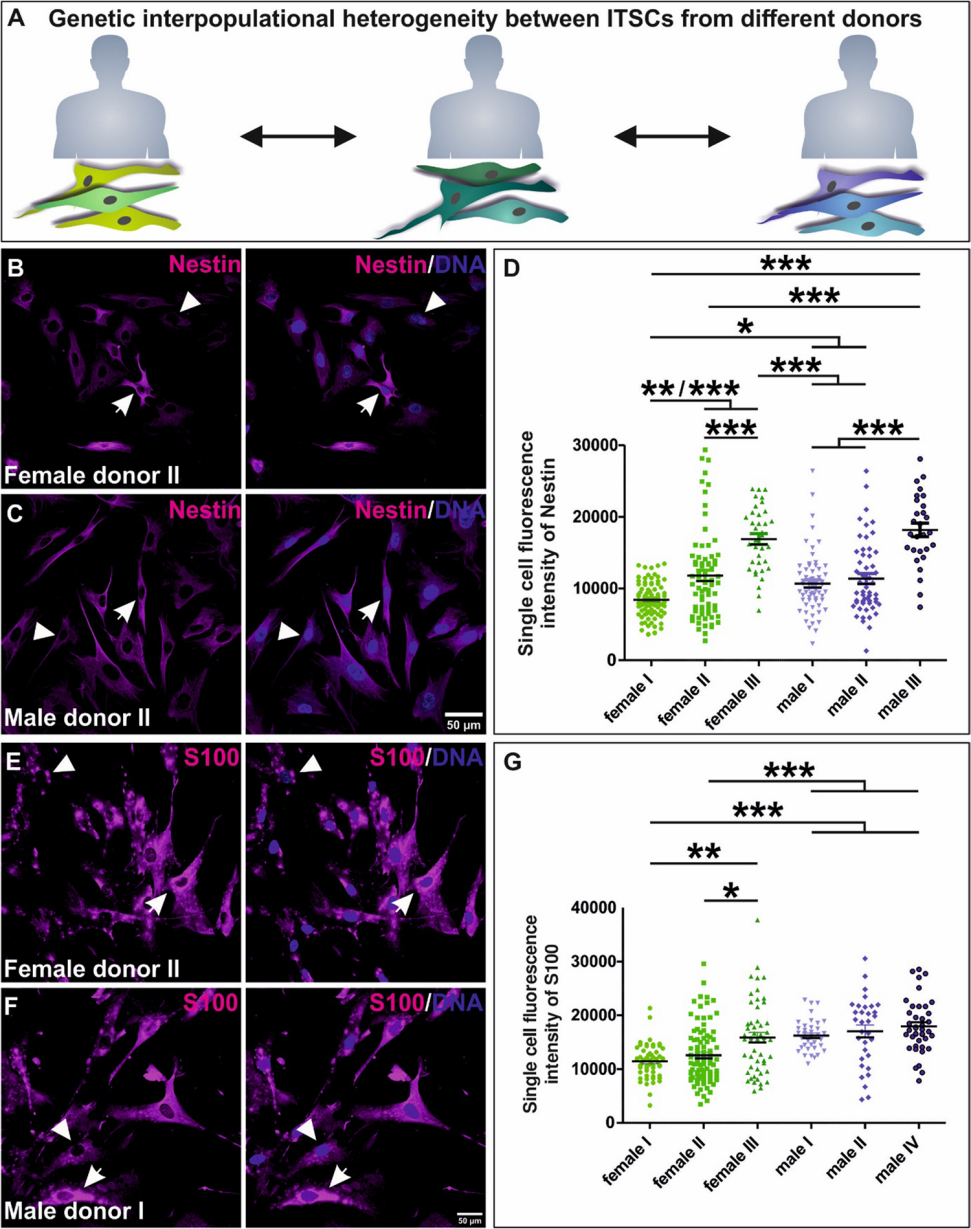
Immunocytochemistry confirmed heterogeneous expression of Nestin and S100 between ITSCs in dependence to the donor. **A**: Schematic view on interpopulational heterogeneity in stem cell marker expression between individual ITSC-populations in dependence to the donor. **B**-**C**: Representative images of immunocytochemical stained Nestin protein in female and male-derived ITSCs. **D**: Quantification of immunocytochemistry via measurement of the cytosolic fluorescence intensity of Nestin per single cell validated a significant heterogeneity dependent on the donor on protein level. **E**-**F**: Representative images of immunocytochemical stained S100 protein in female and male-derived ITSCs. **G**: Quantification of immunocytochemistry via measurement of the cytosolic fluorescence intensity of S100 per single cell affirmed the heterogeneity of S100 expression on protein level. Kruskal-Wallis test, Post test: Dunn’s Multiple Comparison Test, *p<0.05, *p<0.01, *** p < 0.001. was considered significant

To investigate if the heterogeneous expression of neural crest and stem cell markers in single ITSCs observed on mRNA level is also present on protein level, immunocytochemistry against Nestin and S100 was performed using ITSCs isolated from three male and three female donors (Fig. 2A). Even though, most of the analyzed single cells did not reveal the presence of the transcript of Nestin (Fig. 1K-L), the intermediate filament and stem cell marker displayed a strong heterogeneity on protein level within the respective ITSC-populations (Fig. 2B-C). Single ITSCs differed between low (Fig. 2B-C, arrowheads) and high (Fig. 2B-C, arrows) amounts of Nestin protein. Notably, we nevertheless observed significant differences in the amount of Nestin protein between ITSC-populations from distinct donors in a sex-independent manner (Fig. 2D). A quantification of the fluorescence intensities also revealed intrapopulational variations in the amount of the neural crest-related protein S100, which was further shown to be significantly varying between ITSC-populations in dependence to the donor (Fig. 2E-G).

In conclusion, we observed a great donor-dependent heterogeneity in the amounts of Nestin and S100 protein between ITSC-populations, although protein amounts likewise strongly varied between single ITSCs.

### Variations in Nuclear Localized Slug and NF-κB-p65 Proteins are Present Between ITSCs on Intrapopulational and Interpopulational Level with Regard to the Donor

**Fig. 3:**
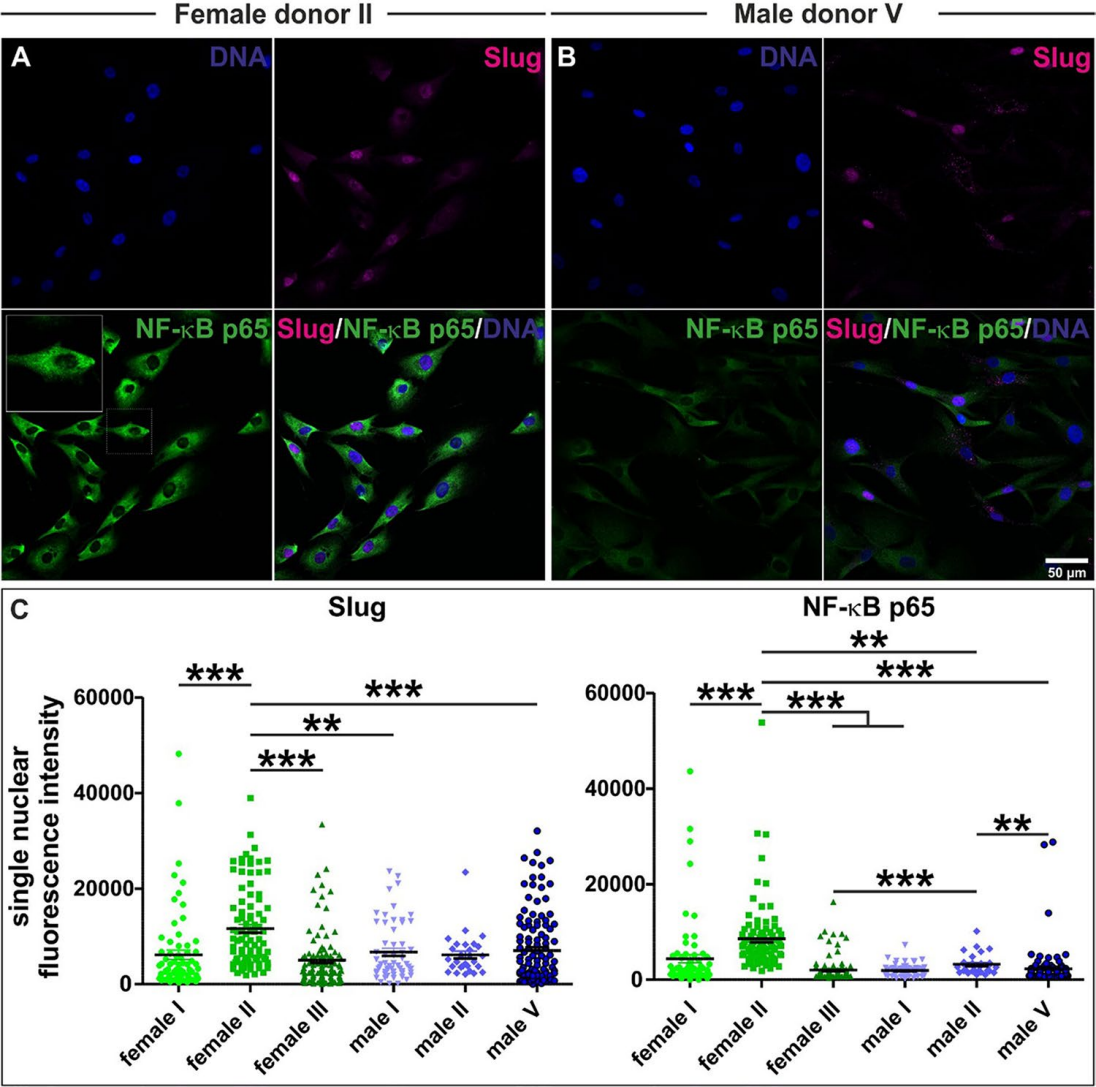
Intra- and interpopulational heterogeneity of NF-κB-p65 and the EMT transcription factor Slug. **A**-**B**: Representative images of immunocytochemical stainings showing heterogeneity of Slug and NF-κB-p65 protein between single ITSCs and ITSC-populations. C: Quantification of immunocytochemical stainings via measurement of the nuclear fluorescence intensity of Slug and NF-κB-p65 per single cell revealed an intrapopulational and interpopulational variability with regard to the donor. Kruskal-Wallis test, Post test: Dunn’s Multiple Comparison Test, *p<0.05, *p<0.01, *** p < 0.001. was considered significant

Assessing potential variations of regulatory NC-related transcription factors like Slug in ITSCs, we observed a strong heterogeneity between single ITSCs within a distinct population regarding the amount of nuclear localized Slug protein (Fig. 3A-B). Measurements of the nuclear fluorescence intensities further revealed a significant heterogeneity between cells of the same ITSC- donor as well as between cells of different ITSC-donors in a sex-independent manner (Fig. 3C). This observation stands in line with the already described transcriptional heterogeneity for the neural crest marker Slug (Fig. 1F-K). With regard to the already described regulation of Slug by NF-κB [28] and its very recently described role in differentiation of ITSCs [24], we also determined NF-κB-p65 nuclear protein amounts in single ITSCs. Likewise to our observation regarding Slug protein, we observed a strong intra- and interpopulational heterogeneity of NF-κB-p65 protein amounts between ITSCs (Fig. 3A-C). Interestingly, the ITSC-population isolated from female donor II showing a significantly increased nuclear protein amount of Slug also revealed a significantly elevated level of nuclear localized NF-κB-p65 (Fig. 3C). These findings are in line with the recently described correlation between the activity of NF-κB-p65 and Slug [28].

### Heterogeneous Protein Amounts of Neural Crest and Stem Cell Markers are also Observable Between Single ITSCs within Clonally Grown Cultures

**Fig. 4:**
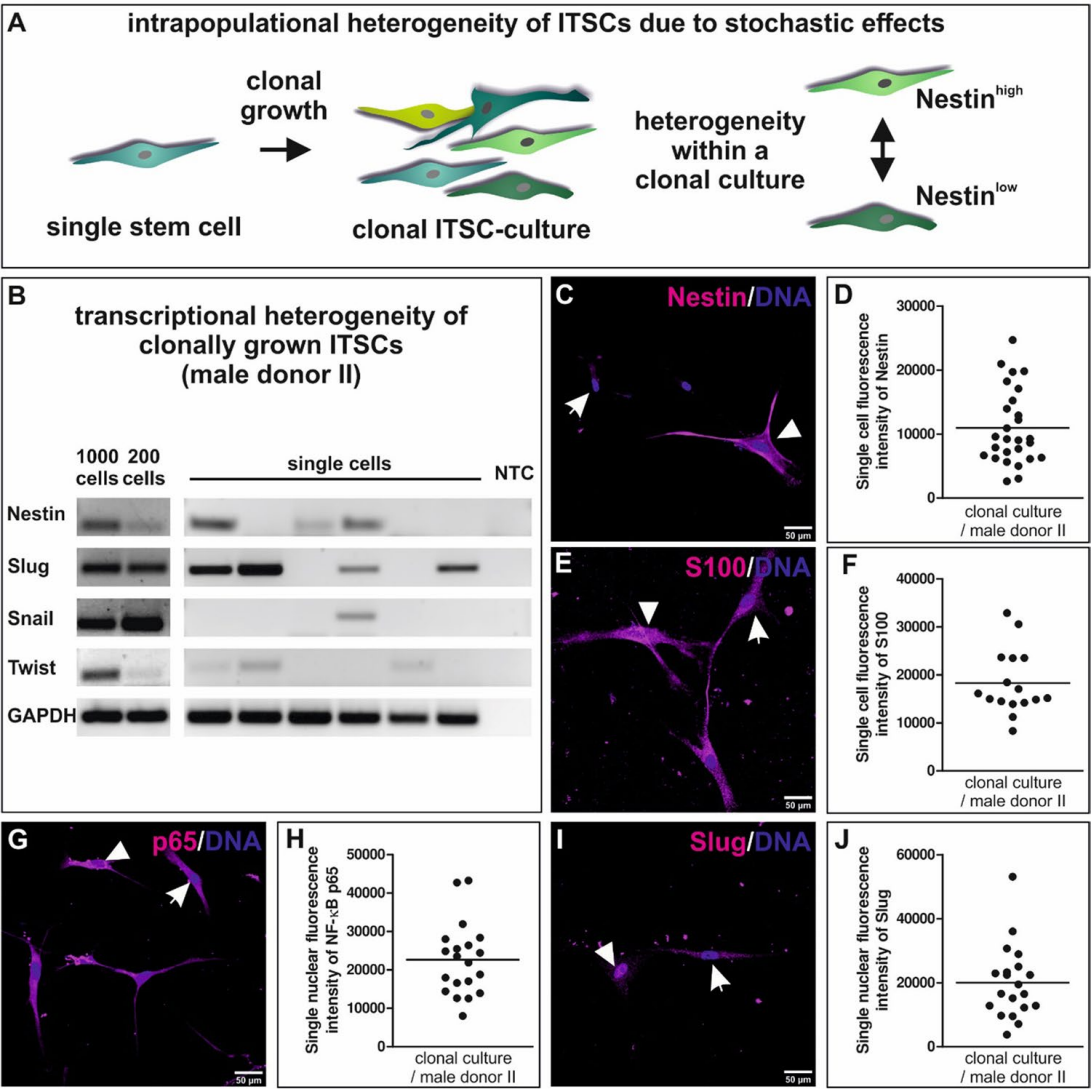
Stochastic single cell heterogeneity within clonally grown ITSC-culture. **A**: Schematic view on heterogeneous protein amounts between single ITSCs in the same clonal culture. **B**: RT-PCR revealing transcriptional heterogeneity of clonally grown ITSCs regarding the expression of NCSC-markers Nestin, Slug, Snail and Twist. **C**, **E**, **G**, **I**: Representative images of immunocytochemical stainings showing heterogeneity of Nestin, S100, Slug and NF-κB-p65 proteins between single ITSCs present in the same clonally grown culture. **D**, **F**, **H**, **J**: Quantification of immunocytochemical stainings via measurement of the single cell or single nuclear fluorescence intensity showing the stochastic variability in Nestin, S100, Slug and NF-κB-p65 protein amounts in clonally grown ITSCs

To assess the origins of the heterogeneous expression of neural crest and stem cell markers observed between single ITSCs, we analyzed transcriptional expression and protein amounts of different stem cell and neural crest markers in clonally grown ITSCs (Fig. 4A). Contrary to our assumption of a more homogenous distribution, transcriptional profiling of clonally grown single ITSCs revealed heterogeneity in the presence of the transcripts for Nestin, Slug, Snail and Twist, too (Fig. 4B). Heterogeneity was further validated on protein level, as the quantification of the immunofluorescence intensities on single cell level revealed highly variable protein levels of the stem cell marker Nestin between ITSCs in the same clonal culture (Fig. 4C-D). Although the NCSC-marker S100 was expressed in high amounts within all analyzed ITSCs of the clonal culture, the protein amount differed between single cells (Fig. 4E-F). Moreover, nuclear fluorescence intensity of Slug and NF-κB-p65 showed highly variable degrees of nuclear translocation on single cell level between ITSCs within a clonal population (Fig. 4G-J). In summary, we provide evidence for a strong heterogeneity even between single ITSCs in the same clonal culture regarding the transcript and protein levels of neural crest and stem cell markers as well as of the subunit p65 of the NF-κB family.

## Discussion

The present study reveals a new perspective on the heterogeneity of adult human neural crest-derived stem cells by demonstrating donor-dependent interpopulational variations in NCSC-marker genes, which are accompanied by an intrapopulational heterogeneity even within clonal cultures.

On the level of donor-to-donor variabilities, we detected variations in the presence of transcripts for the stem cell and neural crest markers Nestin, Slug, Snail and Twist. We further found the protein amounts of Nestin, S100 and Slug to be significantly different between populations of NC-derived inferior turbinate stem cells from distinct donors. In accordance to our present observations, Clewes and colleagues reported a donor-dependent molecular signature of epidermal neural crest-derived stem cells, particularly including pluripotency-associated genes [6]. Although the age of the donor is often related to variations in the behavior of adult stem cells (reviewed in [29]), we could not observe an association between donor age and the heterogeneous protein amounts of Nestin, S100, Slug or NF-κB. In particular, female donors of similar age (female donor II: 67 y, female donor III: 61 y) revealed a significant difference in the amounts of Nestin, S100, Slug and NF-κB protein. In addition, the interpopulational heterogeneity in marker expression on transcriptional and protein level shown here was not attributed to potential sexual dimorphisms, which are increasingly noticed to drive heterogeneous behavior of NCSCs and other adult stem cells ([16] reviewed in [30]). We therefor suggest genetic variations between the distinct donors to account for the observed differences in NCSC-marker expression between the analyzed ITSC-populations, which will be assessed in more detail in future work.

Next to the donor-dependent variations between distinct ITSC-populations, we even observed a great intrapopulational heterogeneity of NCSC-marker protein amounts within the analyzed populations. In accordance to our findings, a range of studies reported intrapopulational variations of NCSCs, namely between clonally grown NCSC-cultures from the same population. The NCSC-clones were shown to strongly vary in their differentiation potential and molecular profile (reviewed in [2]). In particular, Singhatanadgit and coworkers reported a highly heterogeneous differentiation potential between clonal human periodontal ligament stem cell cultures, which varied from sole osteogenic to multilineage differentiation [31]. Human dental pulp stem cell (DPSC) clones were further reported to reveal a great variability in the potential for generating dentin *in vitro* and *in vivo* [15]. In previous studies, we also observed clonal ITSC-cultures to reveal different ratios of ectodermal to mesodermal progeny upon spontaneous differentiation [13, 19]. Linking this genetic heterogeneity in differentiation potential of NCSCs to heterogeneous gene expression levels of NCSC-markers, Young and colleagues demonstrated Nestin to be highly differentially expressed between murine DPSC-clones. The authors further observed a high expression of Nestin to be a pre-requisite for neuronal and oligodendrocyte differentiation [32]. Extending these promising findings, our present results show strong transcriptional heterogeneity for Nestin, Slug, Snail and Twist as well as highly heterogenous protein amounts of Nestin, S100, Slug and NF-κB-p65 even between single ITSCs in the same clonal culture. In addition to intrinsic genetic variations among clonal cultures, these observations suggest stochastic heterogeneity to at least partly account for the variability in NCSC-marker expression shown between single ITSCs. Stochastic variations in transcription or cell cycle have been already shown to account for heterogeneous transcription factor activity, molecular profiles and behavior of embryonic stem cells (ESCs) [33–35]. Such stochastic transcriptional heterogeneity of ESCs can be particularly based on histone modifications [36], allele-switching [34], mRNA half-life [34] and transcriptional bursting, the stochastic activation and inactivation of promoters [37], which is in turn driven by a range of promoter- and gene body-binding proteins [38]. Post-transcriptional variations in protein synthesis and degradation further contribute to the regulation of heterogeneous stem cell identities and enable stem cell homeostasis [39]. Interestingly, we detected discrepancies between the heterogeneous transcript and protein levels for the NCSC-marker Nestin in the present study, suggesting the occurrence of transcriptional bursting events, a short mRNA half-life as well as and/or slow degradation rates of Nestin protein. In addition to ESCs, heterogeneity of hematopoietic stem cells (HSCs) is also controversially discussed to be partly driven by stochastic variations [40–42]. Stochastic fluctuations in transcription as discussed above as well as bistable epigenetic states are also suggested to build the basis of intraclonal heterogeneity observed in mesenchymal stem cells (MSCs) [43](reviewed in [29]). Despite the well-studied basis of stochastic heterogeneity in ESCs and their potential role in adult HSCs and MSCs, similar mechanisms remain to be investigated in more detail in human neural crest-derived stem cells. The present study provides evidence for the first time that intrapopulational heterogeneity of NC-derived human ITSCs is at least partly based on stochastic variations in NCSC-markers, which may include variations in cell cycle or transcription as discussed above. We propose this stochastic intrapopulational heterogeneity to occur in addition to the already described genetic variability between clonal NCSC-cultures and the niche-dependent plasticity of NCSCs (reviewed in [2]). Off note, extrinsic stimuli related to the culture condition may be suggested to likewise account for the heterogeneous expression of NCSC-markers. However, the cultivation method utilized here was previously demonstrated to assure genetic stability and stemness of ITSCs [19], thus minimizing potential extrinsic influences. Moreover, we recently observed an intrapopulational heterogeneity within the migration behavior of NC-derived hCSCs, even when utilizing a microfluidic cultivation system to minimize culture-dependent extrinsic stimuli [44].

On regulatory level, our data show an increased nuclear protein amount of Slug in ITSCs from one female donor, which was accompanied by a significantly elevated level of nuclear NF-κB-p65 protein, suggesting a NF-κB-dependent regulation of Slug. In this line, Zhang and coworkers reported Slug to be directly regulated by NF-κB in the early vertebrate mesoderm, while Slug upregulation in turn indirectly drove NF-κB RelA expression [45]. Slug belongs to a group of transcription factors also including amongst others Snail and Twist, which are required for neural crest formation and enable EMT and therefore migration of embryonic NCCs [45, 46]. For adult NCSCs, we recently suggested such NC-related transcription factors as Slug as well as NF-κB to be vital for maintaining stemness (reviewed in [2]). Accordingly, Tang and coworkers demonstrated Snail and Slug to cooperatively control self-renewal and differentiation of adult stem cells from the murine bone marrow [47]. We further recently reported inhibition of NF-κB c-Rel during differentiation of ITSCs to result in a fate shift from the neuronal to oligodendroglial lineage [24]. The present data extend these promising findings by demonstrating heterogeneous mRNA expression levels and protein amounts of these core NCSC-markers as well as NF-κB on an inter- and intrapopulational level, suggesting the presence of heterogeneous NCSC-stemness states. Accordingly, Gadye and coworkers reported the presence of transient stemness states in murine NCSCs residing in the olfactory epithelium following injury [48].

In summary, we show a donor-dependent interpopulational variation in NCSC-marker expression between ITSC-populations from distinct donors on transcriptional and posttranscriptional level. This interpopulational heterogeneity was accompanied by stochastic intrapopulational differences in NCSC-marker expressions on protein and transcript levels between clonally grown single ITSCs. We consider these stochastic intrapopulational differences as an additional level of heterogeneity to the already described genetic heterogeneity between clonal NCSC-cultures and their niche-dependent plasticity. Our present findings thus offer a broader perspective on NCSC-heterogeneity and may serve to better understand NCSC-behavior.

## Data Availability

All data are made available within the manuscript.
